# Supplementary material for: Blood DNA Methylation in Nuclear and Mitochondrial Sequences Links to Malnutrition and Poor Prognosis in ALS: A Longitudinal Study
Source: Nutrients. 2025 Apr 8;17(8):1295. doi: 10.3390/nu17081295 (PMC12030252; doi:10.3390/nu17081295)
Supplement: Supplementary file 1 [file nutrients-17-01295-s001.zip › nutrients-3513998-supplementary.pdf]

Table S1. Primer sequences used in the pyrosequencing analysis in the present study.

Gene Name	Amplicon size (bp)	Number of CpG sites	Primer Name	Sequence 5′ > 3′
D-loop	226	4	D-loop pre-PCR forward	[Btn] TAGGATGAGGTAGGAATTAAAGATAGATA
			D-loop pre-PCR reverse	ACATCTAATTCCTACTTCAAAATCAT
			D-loop sequencing	CAAATCTATCACCCTATTAA
GSTP1	204	2	GSTP1 pre-PCR forward	GAGTTCGCGGGATTTTTTAGAAGAG
			GSTP1 pre-PCR reverse	[Btn] ACTACCAATTAACCCCATACTAAAAACTCT
			GSTP1 sequencing	GGGCGGGATTATTTTTATAAGG
LINE-1	308	4	LINE-1 pre-PCR forward	TTTTGAGTTAGGTGTGGGATATA
			LINE-1 pre-PCR reverse	[Btn] AAAATCAAAAAATTCCCTTTC
			LINE-1 sequencing	AGTTAGGTGTGGGATATAGT

Table S2. Differences in DNA methylation levels of studied sequences depending on nutritional, progression and survival status in patients with amyotrophic lateral sclerosis (ALS)

Nutritional Status - GLIM Criteria													
Genes - CpG sites	No malnutrition (n=35)		Moderate Malnutrition (n=15)		Severe Malnutrition (n=13)		p-value	p-value	Adjusted p-value	p-value	Adjusted p-value	p-value	Adjusted p-value
	Mean (SD)	Median (1st,3rd Q)	Mean (SD)	Median (1st,3rd Q)	Mean (SD)	Median (1st,3rd Q)		NoM-MM	NoM-MM	NoM-SM	NoM-SM	MM-SM	MM-SM
**D-loop_CpG1**	2.63 (1.68)	2 (2,3)	2.27 (0.59)	2 (2,2)	1.92 (0.28)	2 (2,2)	0.0470	0.3255	0.9764	0.0141	0.0422	0.1923	0.5770
**D-loop_CpG2**	2.37 (0.77)	2 (2,3)	2.13 (0.64)	2 (2,2)	1.46 (0.66)	2 (1,2)	0.0006	0.2833	0.8499	0.0001	0.0003	0.0148	0.0444
**D-loop_CpG3**	2.37 (4.62)	1(1,1)	1.13 (0.52)	1 (1,1)	1.00 (0.00)	1 (1,1)	0.2070	0.2849	0.8548	0.0964	0.2892	0.5794	>0.9999
**D-loop_CpG4**	1.83 (2.54)	1(1,2)	1.20 (0.68)	1 (1,1)	0.92 (0.28)	1 (1,1)	0.0146	0.1187	0.3560	0.0052	0.0155	0.2602	0.7806
**D-loop_Mean CpGs**	**2.30 (1.39)**	**1.8 (1.5, 2.5)**	**1.63 (0.55)**	**1.5 (1.5, 1.8)**	**1.34 (0.24)**	**1.5 (1.1, 1.5)**	**0.0001**	**0.0053**	**0.0159**	**0.0001**	**0.0004**	**0.3115**	**0.9344**
**GSTP1_CpG1**	1.43 (1.01)	1(1,2)	1.60 (0.83)	1 (1,2)	2.54 (1.51)	2 (2,4)	0.0227	0.5100	>0.9999	0.0060	0.0180	0.0689	0.2067
**GSTP1_CpG2**	1.34 (0.80)	1(1,2)	1.73 (0.88)	2 (1,2)	2.15 (1.14)	2 (1,3)	0.0434	0.1916	0.5748	0.0155	0.0464	0.3116	0.9347
**GSTP1_Mean CpGs**	**1.39 (0.83)**	**1 (1,2)**	**1.67 (0.79)**	**1.5 (1,2)**	**2.35 (1.31)**	**2 (1, 4)**	**0.0292**	**0.2899**	**0.8698**	**0.0083**	**0.0249**	**0.1613**	**0.4839**
**LINE-1_CpG1**	71.91 (2.69)	72 (70,73)	71.60 (3.58)	73 (69,74)	74.00 (1.96)	73 (73,75)	0.0260	0.7846	>0.9999	0.0079	0.0238	0.0401	0.1202
**LINE-1_CpG2**	65.34 (7.67)	67 (64,69)	65.47 (3.54)	66 (63,68)	67.46 (2.90)	68 (66,70)	0.3301	0.3456	>0.9999	0.4035	>0.9999	0.1377	0.4132
**LINE-1_CpG3**	67.34 (4.84)	68 (64,70)	65.67 (3.90)	66 (63,68)	68.23 (3.03)	68 (67,71)	0.1765	0.1624	0.5772	0.3662	>0.9999	0.0663	0.1990
**LINE-1_CpG4**	73.57 (4.27)	74 (70,76)	72.73 (3.37)	72 (71,75)	74.31 (2.46)	75 (72,76)	0.4270	0.4021	>0.9999	0.4717	>0.9999	0.1938	0.5315
**LINE-1_Mean CpGs**	**69.57 (3.80)**	**71 (67,73)**	**69.33 (3.54)**	**70 (67,72)**	**70.52 (2.00)**	**70 (69,72)**	**0.8340**	**0.6754**	**>0.9999**	**0.7672**	**>0.9999**	**0.5520**	**>0.9999**
GLIM: Global Leadership Initiative on Malnutrition; NoM: No malnutrition; MM: Moderate Malnutrition; SM: Severe Malnutrition.													

Progression Status of ALS - ALSFRS-R													
Genes - CpG sites	Slow (n=35)		Moderate (n=15)		Fast (n=13)		p-value	p-value	Adjusted p-value	p-value	Adjusted p-value	p-value	Adjusted p-value
	Mean (SD)	Median (1st,3rd Q)	Mean (SD)	Median (1st,3rd Q)	Mean (SD)	Median (1st,3rd Q)		S-M	S-M	S-F	S-F	M-F	M-F
**D-loop_CpG1**	2.71 (1.58)	2 (2,3)	2.00 (0.93)	2 (2,2)	2.00 (0.41)	2 (2,2)	0.0211	0.0296	0.0887	0.0229	0.0686	0.8583	>0.9999
**D-loop_CpG2**	2.20 (0.53)	2 (2,2)	2.27 (0.88)	2 (2,3)	1.77 (1.17)	1 (2,2)	0.0422	0.9414	>0.9999	0.0150	0.0451	0.1046	0.1288
**D-loop_CpG3**	2.37 (4.63)	1 (1,1)	1.07 (0.26)	1 (1,1)	1.08 (0.28)	1 (1,1)	0.6114	0.3909	>0.9999	0.4703	>0.9999	0.9364	>0.9999
**D-loop_CpG4**	1.46 (0.66)	1 (1,2)	2.07 (3.88)	1 (1,1)	0.92 (0.28)	1 (1,1)	0.0153	0.1260	0.3781	0.0053	0.0159	0.2529	0.7588
**D-loop_Mean CpGs**	**2.19 (1.32)**	**1.8 (1.5,2.3)**	**1.85 (1.02)**	**1.5 (1.5,1.8)**	**1.44 (0.33)**	**1.5 (1.3,1.5)**	**0.0052**	**0.1247**	**0.3742**	**0.0015**	**0.0045**	**0.1412**	**0.4236**
**GSTP1_CpG1**	1.54 (1.12)	1 (1,2)	2.00 (1.41)	2 (1,2)	1.77 (0.93)	2 (1,2)	0.4342	0.2571	0.7713	0.3590	>0.9999	0.8912	>0.9999
**GSTP1_CpG2**	1.46 (0.95)	1 (1,2)	1.87 (1.06)	2 (1,2)	1.69 (0.75)	2 (1,2)	0.3424	0.1849	0.5547	0.3304	0.9911	0.8061	>0.9999
**GSTP1_Mean CpGs**	**1.50 (0.96)**	**1 (1,2)**	**1.93 (1.21)**	**1.5 (1,2)**	**1.73 (0.81)**	**1.5 (1,2)**	**0.3403**	**0.1830**	**0.5490**	**0.3308**	**0.9924**	**0.8019**	**>0.9999**
**LINE-1_CpG1**	71.60 (2.64)	72 (70,73)	72.13 (3.46)	72 (70,74)	74.23 (2.09)	73 (73,76)	0.0121	0.4191	>0.9999	0.0030	0.0089	0.0589	0.1767
**LINE-1_CpG2**	65.97 (3.71)	67 (63,69)	64.40 (11.04)	68 (64,69)	67.00 (2.83)	67 (66,70)	0.7244	0.4837	>0.9999	0.5647	>0.9999	0.9388	>0.9999
**LINE-1_CpG3**	66.40 (3.84)	66 (63,69)	67.00 (3.32)	67 (64,69)	69.23 (6.04)	68 (67,71)	0.4650	0.6347	>0.9999	0.2184	0.6552	0.5041	>0.9999
**LINE-1_CpG4**	73.26 (4.43)	73 (70,76)	73.73 (2.87)	75 (72,75)	74.00 (2.58)	74 (72,76)	0.6704	0.5163	>0.9999	0.4384	>0.9999	0.8921	>0.9999
**LINE-1_Mean CpGs**	**69.31 (3.31)**	**70 (67,71)**	**69.32 (4.33)**	**71 (67,73)**	**71.12 (2.19)**	**71 (70,73)**	**0.2855**	**0.5713**	**>0.9999**	**0.1144**	**0.3432**	**0.3723**	**>0.9999**

ALS: amyotrophic lateral sclerosis; ALSFRS-R: Revised Amyotrophic Lateral Sclerosis Functional Rating Scale; S: Slow Progression; M: Moderate Progression; F: Fast Progression**.**

Survival of patients with ALS

**Genes - CpG sites**	**Survival** (n=35)		**Death** (n=28)		p-value	Adjusted p-value
	Mean (SD)	Median (1st,3rd Q)	Mean	Median (1st,3rd Q)	S-D	S-D
**D-loop_CpG1**	2.63 (1.66)	2 (2,3)	2.11 (0.57)	2 (2,2)	0.0432	0.1620
**D-loop_CpG2**	2.26 (0.61)	2 (2,2)	1.96 (0.96)	2 (1,2)	0.0433	0.1620
**D-loop_CpG3**	2.34 (4.63)	1 (1,1)	1.11 (0.42)	1 (1,1)	0.2893	0.2893
**D-loop_CpG4**	1.74 (2.54)	1 (1,2)	1.18 (0.67)	1 (1,1)	0.1158	0.2182
**D-loop_Mean CpGs**	**2.24 (1.40)**	**1.8 (1.5,2.3)**	**1.59 (0.50)**	**1.5 (1.3,1.7)**	**0.0028**	**0.0033**
**GSTP1_CpG1**	1.46 (1.01)	1 (1,2)	2.00 (1.28)	2 (1,3)	0.0889	0.0889
**GSTP1_CpG2**	1.34 (0.80)	1 (1,2)	1.93 (1.02)	2 (1,2)	0.0216	0.0428
**GSTP1_Mean CpGs**	**1.40 (0.83)**	1 (1,2)	**1.96 (1.11)**	1.8 (1,2)	**0.0436**	**0.0446**
**LINE-1_CpG1**	72.09 (3.18)	72 (70,74)	72.50 (2.56)	73 (72,74)	0.2867	0.7411
**LINE-1_CpG2**	65.06 (7.61)	67 (64,69)	66.75 (3.37)	67 (64,70)	0.4832	0.8620
**LINE-1_CpG3**	67.17 (4.95)	68 (64,70)	67.07 (3.54)	67 (64,69)	0.9862	0.9862
**LINE-1_CpG4**	73.40 (4.26)	74 (70,76)	73.68 (3.04)	74 (72,76)	0.6260	0.8620
**LINE-1_Mean CpGs**	**69.43 (3.89)**	**71 (67,73)**	**70.00 (2.78)**	**70 (69,72)**	**0.9043**	**0.9063**

ALS: amyotrophic lateral sclerosis; S: Survival; D: Death.
